# Regeneration of deactivated activated carbon honeycomb catalysts for VOC removal: structural evolution and activity recovery mechanisms

**DOI:** 10.1039/d6ra04608h

**Published:** 2026-07-22

**Authors:** Hangchang Huang, Xinggang Tan, Genlong Li, Shunqiang Chen, Jilong Chen, Sutong Wang, Kaiwen Ren, Xianbin He, Wenjie Qiu, Huibin Guo

**Affiliations:** a Fujian Xinyeindustrial and Management Group Co., Ltd. Xiamen 361026 China; b Xiamen Yuchun Environmental Protection Technology Co., Ltd. Xiamen 361101 Fujian China; c Department of Environmental Engineering, Xiamen University of Technology Xiamen 361024 China guohb@xmut.edu.cn

## Abstract

The deactivation problem of honeycomb catalysts used for industrial volatile organic compound treatment will greatly restrict their long-term effectiveness and lower the overall economic benefits. The long-term operational data shows that the adsorption efficiency has gradually decreased from the original 90 to 95% to 82 to 88%, accompanied by greater fluctuations and more low efficiency situations, indicating that the catalyst is gradually becoming deactivated. In this study, a regeneration process including physical cleaning, chemical washing, and thermal treatment was used for deactivated activated carbon honeycomb catalysts. After regeneration, the catalyst structure and catalytic performance were recovered. BET results showed that the specific surface area increased from 29.57 to 54.44 m^2^ g^−1^, and the micropore area increased from 11.85 to 25.90 m^2^ g^−1^. SEM images showed that surface deposits were removed and blocked pore channels were reopened. XRD results indicated that the carbon framework and active phases remained structurally stable during regeneration. XPS results demonstrated variations in surface chemical composition, including the partial removal of sulfur- and chlorine-containing deposits, while maintaining the overall carbon framework. Furthermore, TGA analysis confirmed a decrease in thermally stable carbonaceous residues after regeneration, indicating the removal of accumulated organic contaminants. Based on the structural characterization results, a deactivation-regeneration mechanism involving contaminant deposition, pore blockage, active site shielding, and pore reopening was proposed. The reconstructed hierarchical pore structure significantly improved pore accessibility and re-exposed active sites, resulting in the recovery of catalytic performance. These research results can provide reliable regeneration methods for honeycomb catalysts used in the treatment of volatile organic compounds in industry.

## Abstract 2

The deactivation problem of honeycomb catalysts used for industrial volatile organic compound treatment will greatly restrict their long-term effectiveness and lower the overall economic benefits.

## Graphical abstract

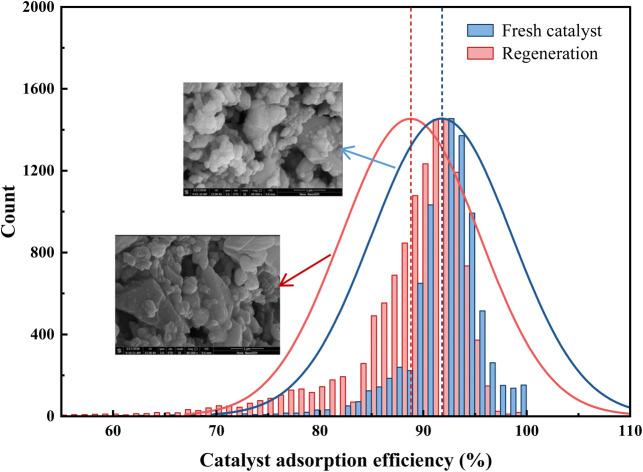

## 1. Introduction

Volatile organic compounds (VOCs) in industry are the main air pollutants. They also pose risks to human health and environmental safety due to their toxicity or the formation of secondary pollutants.^1–3^ Recent studies have further demonstrated that atmospheric organic pollutants can participate in the formation of environmentally persistent free radicals (EPFRs), which contribute to oxidative potential and reactive oxygen species (ROS) generation in particulate matter, thereby increasing the environmental and health risks associated with air pollution. Activated carbon honeycomb catalysts are widely used to eliminate VOCs because of their unique structure, low pressure drop, large specific surface area and good catalytic oxidation performance.^4–6^ Although having advantages, long-term use in complex flue gas will deactivate the catalyst, mainly because the pores are blocked, carbon is deposited, and active sites are poisoned by S, Cl or metal-containing substances.^7–9^

Catalyst deactivation has become one of the main technical problems limiting their industrial application and service life.^10,11^ Existing studies have confirmed that catalyst deactivation is usually associated with several types of situations, including carbon deposition, pore blockage, poisoning caused by sulfur-, chlorine- and phosphorus-containing compounds, high-temperature sintering of active components, and collapse of support structure.^12–15^ In addition, inorganic particulates and metal-containing impurities in industrial exhaust gases may further aggravate pore blockage and surface contamination.^16^ Under high-temperature operating conditions, migration and aggregation of active metal species may occur, leading to reduced dispersion and irreversible activity loss.^17,28,29^ To restore catalytic performance and extend catalyst lifetime, various regeneration technologies have been investigated, including thermal regeneration, chemical washing, ultrasonic cleaning, and combined physical-chemical regeneration methods.^18^ Thermal regeneration can remove adsorbed organic contaminants effectively, but excessive temperatures may induce structural sintering or phase transformation of active components.^19^ Chemical cleaning can dissolve deposited inorganic pollutants and surface poisons, but inappropriate chemical conditions may damage the surface structure of the catalyst or cause the leaching of active components. The three cooperative regeneration steps are physical pretreatment, targeted chemical cleaning and regulated thermal activation. Based on the above studies, such a strategy can address multiple deactivation paths simultaneously and has higher regeneration efficiency than single-step approaches.^20^ Although some progress has been made in the field of related research, the structural alterations during the regeneration of activated carbon honeycomb catalysts and their connection to the recovery of catalytic activity have not yet been fully elucidated. In particular, the dynamic reconstruction of pore structures, surface morphology, and crystal phases during regeneration has not been systematically clarified.^21,22,30^

In this study, the structural evolution and activity recovery mechanisms of activated carbon honeycomb catalysts during regeneration are systematically investigated using a combination of physicochemical characterization techniques, including SEM, TEM, BET, XRD and TGA analyses. The changes in surface morphology, pore structure, and crystalline phases are correlated with catalytic performance to elucidate the intrinsic relationship between structural reconstruction and activity restoration.

## 2. Materials and methods

### 2.1 SEM characterization

The morphology and microstructure of the samples were observed using a field-emission scanning electron microscope (FE-SEM, Hitachi Regulus 8100, Japan).

SEM analysis was conducted to observe the surface morphology and pore structure of the catalysts. Carbon deposition and pore blockage are identified from the images. The regeneration effect is assessed by comparing the surface condition and pore reopening before and after treatment.

### 2.2 TEM characterization

The morphology, particle size, and microstructural characteristics of the catalysts were investigated using transmission electron microscopy (TEM, JEOL JEM-2100 F, Japan). Prior to analysis, the samples were ultrasonically dispersed in ethanol and a small amount of suspension was dropped onto carbon-coated copper grids. TEM images were collected to evaluate the dispersion of active components, particle distribution, and structural changes of the catalysts before and after treatment.

### 2.3 BET surface area measurement

The specific surface area and pore structure of the samples were determined by N_2_ adsorption–desorption measurements using an automated surface area and porosity analyzer (Micromeritics ASAP 2460, USA).

The specific surface area of the samples was determined by N_2_ adsorption–desorption measurements. Approximately 100 mg of catalyst was weighed and degassed under vacuum at 250 °C for 5 h to remove surface adsorbates. The N_2_ adsorption–desorption isotherms were then recorded at 77 K. The specific surface area was calculated using the BET method in the relative pressure (*P*/*P*_0_) range of 0.05–0.30. The total pore volume was obtained from the adsorption amount at *P*/*P*_0_ = 0.99, and the pore size distribution was determined using the BJH model.

### 2.4 XRD analysis

The crystalline structure of the catalysts was characterized by X-ray diffraction (XRD) using a diffractometer (Bruker D8 Advance, Germany) with Cu Kα radiation (*λ* = 1.5406 Å). The data were collected over a 2*θ* range of typically 5–80° at a suitable scanning rate.

### 2.5 XPS analysis

The surface elemental composition and chemical states of the catalysts were characterized by X-ray photoelectron spectroscopy (XPS, Thermo Scientific K-Alpha, USA) equipped with a monochromatic Al Kα X-ray source. The binding energies were calibrated using the C 1s peak at 284.8 eV. Peak fitting analysis was performed to determine the relative contents and chemical states of surface species. XPS analysis was conducted to investigate the variation of surface active species and oxidation states before and after catalyst regeneration.

### 2.6 TGA analysis

The thermal stability and carbon deposition behavior of the catalysts were analyzed by thermogravimetric analysis (TGA, Netzsch STA 449 F5, Germany). Approximately 10 mg of sample was placed in an alumina crucible and heated from room temperature to the target temperature under an air atmosphere at a constant heating rate. The weight loss profiles were recorded to evaluate the amount of carbonaceous deposits and their oxidation behavior. The differences in weight loss before and after regeneration treatment were used to assess the removal efficiency of deposited species.

## 3. Results and discussion

### 3.1 Regeneration procedure

**Fig. 1:**
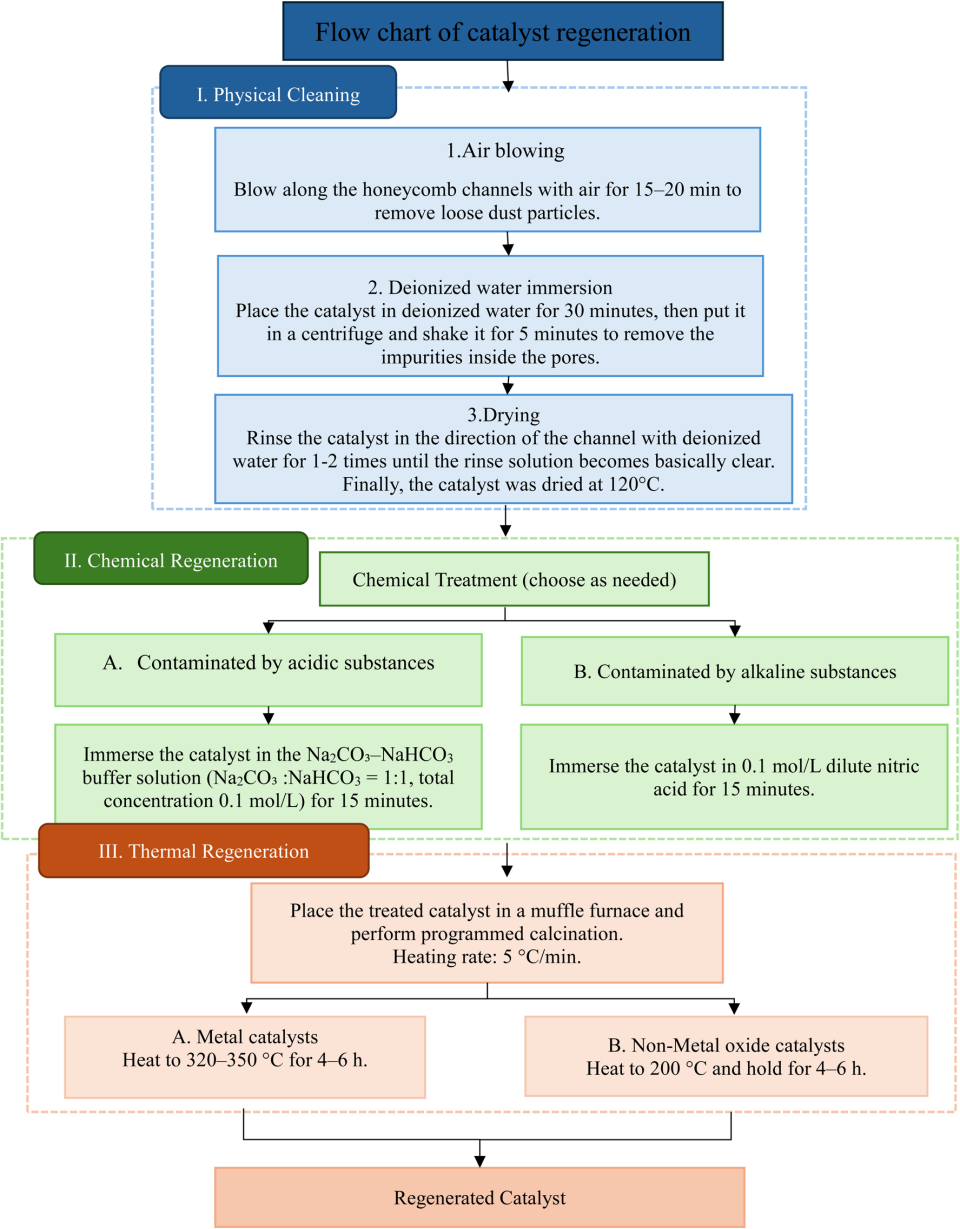
Catalyst regeneration process flow chart.

The entire regeneration process is divided into three steps: physical cleaning, chemical cleaning and heat treatment, as shown in Fig. 1. First, the deactivated catalyst is physically pretreated. Continuously blow compressed air in the direction of the hole for 15–20 minutes to remove loosely attached dust particles. The catalyst was then immersed in deionized water for 30 minutes, during which time it was oscillated and shaken for about 5 minutes to promote the desorption of impurities in the channel. Subsequently, the catalyst was rinsed 1–2 times with deionized water until the wastewater became transparent.

Secondly, chemical regeneration treatment is carried out according to the type of pollutants. For catalysts contaminated by acidic substances such as halogenated hydrocarbons, use Na_2_CO_3_–NaHCO buffer (Na_2_CO_3_ : NaHCO_3_ = 1 : 1, total concentration 0.1 mol L^−1^), immerse the catalyst for about 15 minutes, and then rinse to near neutral pH. For catalysts containing sulfate or nitrate deposits, use 0.1 mol L^−1^ dilute nitric acid for 15 minutes, followed by a thorough rinse with deionized water. After chemical treatment, the catalyst was dried at 120 °C for 2 hours.

Finally, thermal regeneration. Place the catalyst into a muffle furnace and heat it to 200 °C, with a heating rate set to 5 °C min^−1^ and a duration of 4–6 hours. After the calcination is completed, the catalyst is naturally cooled to room temperature.

### 3.2 Catalyst adsorption efficiency

**Fig. 2:**
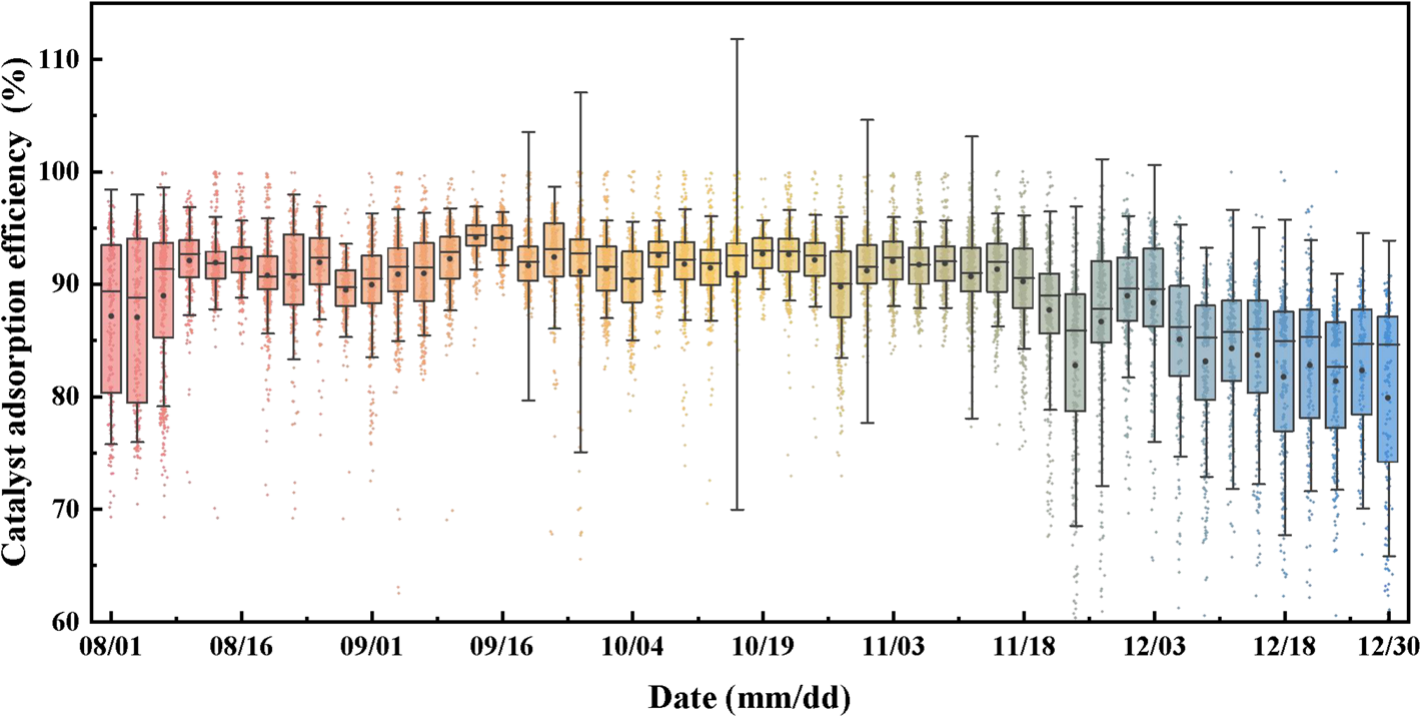
Dynamic changes in adsorption efficiency of catalysts during long-term operation.

Before regenerating the catalyst, its adsorption efficiency was evaluated based on long-term operating data, as shown in Fig. 2.

At the beginning of August, the adsorption efficiency of catalyst was relatively high, with the medium case being about 90–93%. However, the lower quartile of catalyst dropped to about 80–85%, and there was instability in the early stage. From September to October, the efficiency distribution became narrower, most of which were between 90–95%, and the adsorption was relatively stable at that time. It dropped significantly after mid-November, the median effective gradient of catalyst reached about 88–90%. At the end of December, the interquartile range rose, more outliers were below 75%, and there were severe perfusion fluctuations and progressive inactivation.

**Fig. 3:**
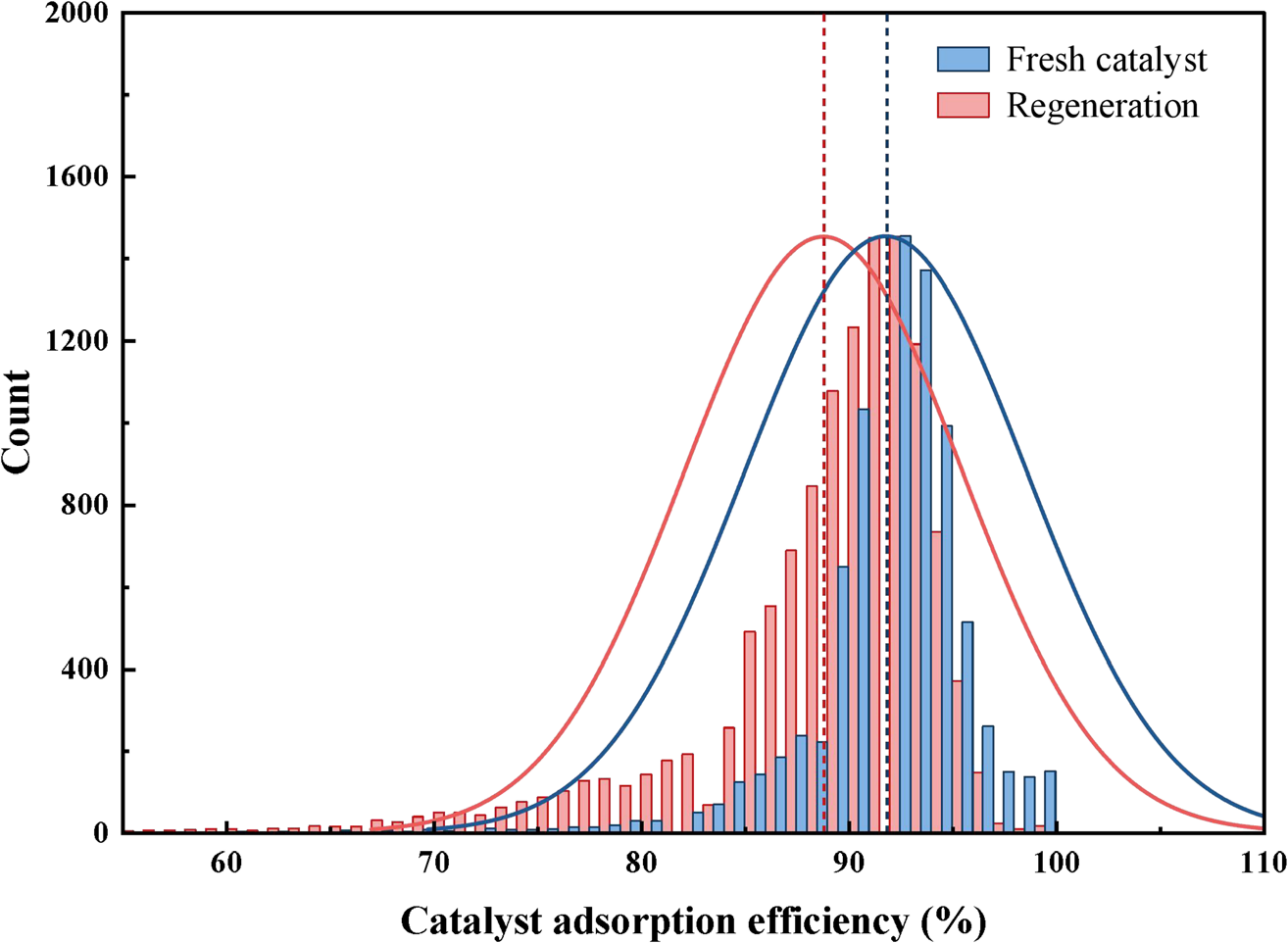
The statistical distribution of catalytic efficiency of fresh catalyst and after regenerated catalyst.

As shown in the Fig. 3, the overall performance of fresh catalyst is significantly better than that of after regenerated catalyst. The average adsorption efficiency of fresh catalyst (*µ* = 91.81%) is notably higher than that of after regenerated catalyst (*µ* = 88.80%), indicating superior overall adsorption performance of fresh catalyst. Meanwhile, the two catalysts exhibit comparable operational stability, as evidenced by their close standard deviations (*σ* = 6.80% for fresh catalyst *vs. σ* = 6.70% for after regenerated catalyst), which suggests no significant difference in efficiency fluctuation levels and similar performance dispersion between the two catalysts. The adsorption efficiency distribution of the regenerated catalyst largely overlaps with that of the fresh catalyst. Although the average adsorption efficiency of the regenerated catalyst is slightly lower, approximately 90% of the adsorption performance of the fresh catalyst is retained, indicating that the regeneration process effectively restores the adsorption capacity.

### 3.3 Morphology characterization

#### 3.3.1 Deactivation characteristics of the catalyst (SEM)

**Fig. 4:**
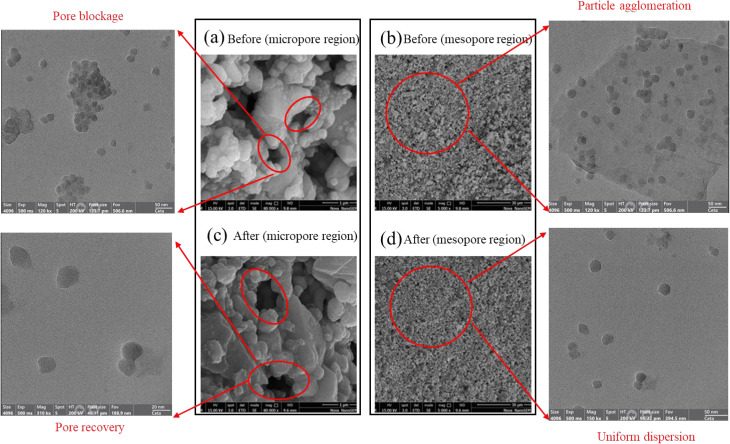
Comparison image of scanning electron microscope (SEM) of catalysts before and after regeneration: images (a and b) correspond to the catalysts before regeneration, while images (c and d) correspond to the catalysts after regeneration.

Scanning electron microscopy, also known as SEM, can be used to observe the changes in surface morphology, pollutant deposition, and pore structure of catalysts during deactivation and regeneration stages.^23^ The author used a scanning electron microscope, also known as SEM, to examine the surface morphology of the deactivated and regenerated activated carbon honeycomb catalyst. The analysis results of the surface morphology of volatile organic pollutants are shown in Fig. 4, the enlarged pictures on the left and right sides are the scenes captured by the TEM experiment.

More than half of the surface of the deactivated catalyst is covered by particulate deposits and carbonaceous substances, which not only blocks many pores but also reduces the number of active sites available. There have been many studies reporting such deactivation in VOC catalytic systems.^12^ During long-term operation, catalysts will gradually accumulate carbon deposits and inorganic impurities on their surface. If we look at the deactivated catalyst, we can see that some particles have agglomerated on the surface, indicating that the catalyst may have sintered in a high-temperature environment. The occurrence of such clustering is consistent with the conclusions of previous related research. High temperature environments can cause migration and aggregation of active ingredients, ultimately leading to a decrease in material dispersion and catalytic activity.^23,24^ Regeneration was carried out; the surface of the catalyst was relatively clean again, and most of the pores had returned to their original state. The sediment on the surface has been removed, and it can also be seen that this way of treating physically and chemically is effective in removing carbon deposits and inorganic impurities. Particle aggregation has decreased; therefore, it can be inferred that the regeneration process has reduced sintering and redistribution of active components to some extent.

According to the detection results of scanning electron microscopy, the reasons for catalyst deactivation mainly fall into two categories: first, the pores have been blocked; second, the surface has been covered by other materials. The regeneration process will increase the number of pores and available catalytic sites in the material and thus improve catalytic performance.

#### 3.3.2 Crystal structure analysis (XRD)

**Fig. 5:**
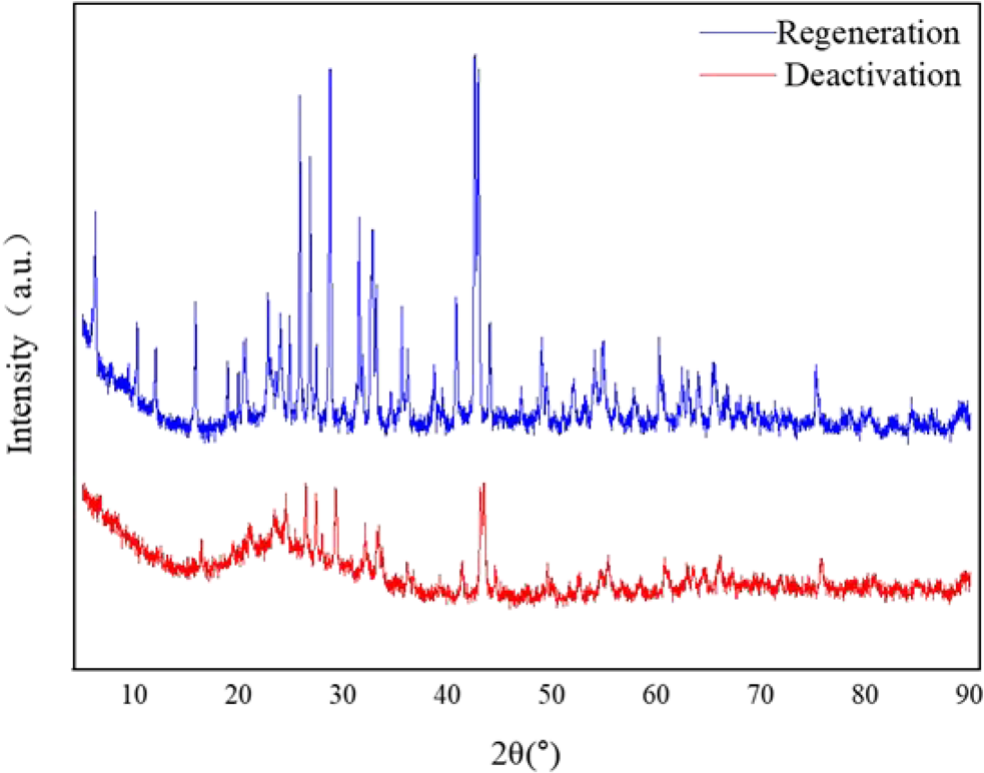
XRD patterns of deactivated, and regenerated catalysts.

XRD was employed to analyze the crystalline structure, phase composition, and structural evolution of the catalysts before and after regeneration.^25^ XRD diffraction analysis of the crystal structure of the deactivated and regenerated catalysts is shown in Fig. 5.

All samples' diffraction patterns have characteristic peaks of active oxide phases; thus, it can be determined that the basic crystal structure after deactivation and regeneration has been retained. The corresponding peaks have different intensities and widths. The average size of the crystals is given by the Scherrer equation:1
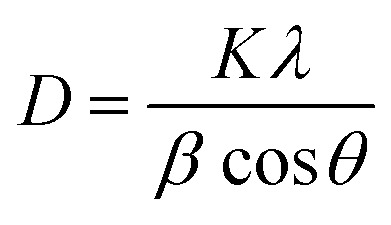
where *D* is the average crystallite size (nm), *K* = 0.89, *λ* is the X-ray wavelength, *β* is the full width at half maximum (FWHM) of the diffraction peak in radians, and *θ* is the Bragg diffraction angle.

The calculated crystallite sizes of the deactivated and regenerated catalysts were 4.94 nm and 7.66 nm, respectively. The new catalyst has a larger crystallite size and a narrower diffraction peak than the deactivated catalyst. Therefore, the regeneration treatment has promoted the growth of crystallites or increased the degree of crystallization in the active phase. It has been shown before that heat can cause a rearrangement and grouping of active sites, leading to the enlargement of crystal domains and a reduction in accessible active sites.^26^ Heat treatment and chemical reaction can be used together in the regeneration stage to promote the rearrangement of active components and thus cause small crystallites to grow into larger, more ordered domains. Although regeneration led to an increase in crystallite size, no extra diffraction peaks of impurity phases were found, and therefore, it was concluded that the regeneration process did not cause phase changes or structural damage to the active metal oxide. Therefore, the active stage was stable and did not change structurally. Based on the above results, catalyst deactivation is due to structural changes in the active phase, and regeneration can restore the structural order and phase stability. However, an increase in crystallite size after regeneration suggests that too high a temperature for too long may cause irreversible sintering and a loss of the active surface area.

The above structural modifications are required to understand how regeneration conditions affect the recovery of catalytic performance.

#### 3.3.3 X-ray photoelectron spectroscopy analysis

**Fig. 6:**
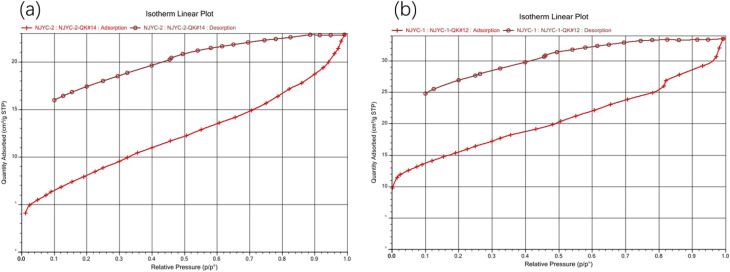
N_2_ adsorption–desorption isotherms of the catalysts before (a) and after (b) regeneration.

XPS was used to study alterations in the surface structure and chemical environment of the catalyst after regeneration by acquiring spectra before and after regeneration. As shown in Fig. 6, the XPS survey spectra indicate that both samples are mainly composed of C and O, and S and Cl have also been detected on the catalyst surface. There are no extra impurity elements after regeneration, so it can be determined that the regeneration process has not caused serious damage to the whole-surface composition of the catalyst.

**Fig. 7:**
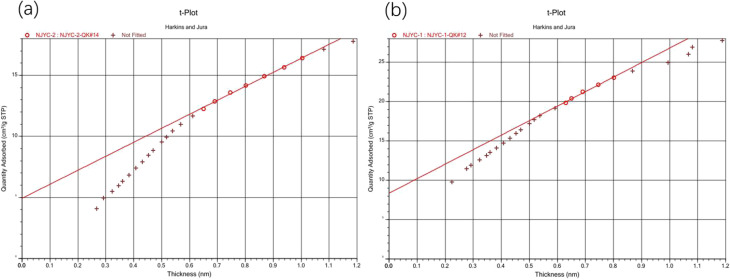
*t*-Plot curves of the catalysts before (a) and after (b) regeneration based on the Harkins–Jura model.

The high-resolution XPS spectra of C 1s, S 2p, and Cl 2p are presented in Fig. 10(a–f). As shown in Fig. 7(a) and (d), the C 1s peak remains centered at approximately 284.8 eV before and after regeneration, with no obvious shift in binding energy, suggesting that the carbon framework remains structurally stable during the regeneration process. The S 2p spectra (Fig. 7(b) and (e)) exhibit similar binding energies before and after regeneration, whereas the peak intensity decreases slightly after regeneration, indicating that sulfur-containing surface deposits are partially removed while a small amount of sulfur species remains on the catalyst surface. Likewise, the Cl 2p spectra (Fig. 7(c) and (f)) show relatively weak signals in both samples, with a slight decrease in peak intensity after regeneration, suggesting the partial elimination of chlorine-containing poisoning species.

Overall, the XPS results demonstrate that the regeneration process does not alter the primary surface composition of the catalyst but effectively removes part of the sulfur- and chlorine-containing deposits, thereby reducing the coverage of active sites and blocked pores. These observations are consistent with the BET and SEM results, which indicate the recovery of the pore structure and the removal of surface deposits after regeneration.

#### 3.3.4 Textural properties analysis (BET)

**Fig. 8:**
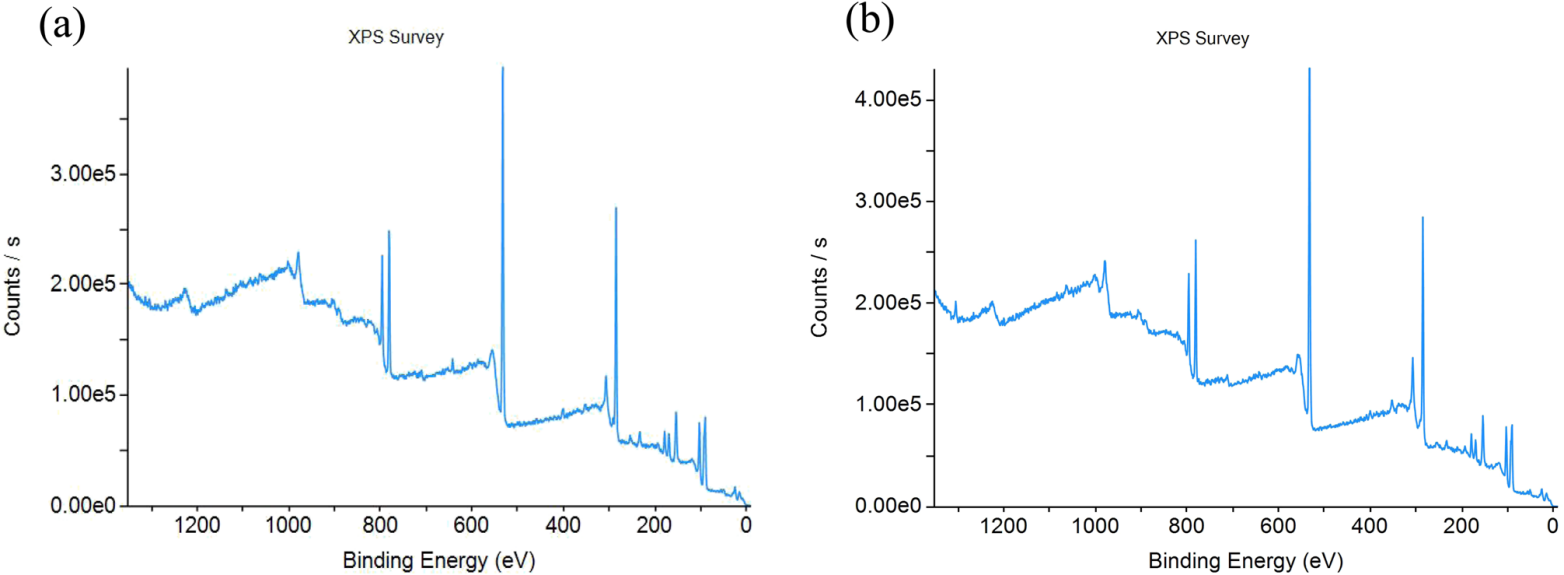
XPS survey spectra of the (a) deactivated catalyst and (b) regenerated catalyst.

The specific surface area, pore structure, and structural changes of the catalyst before and after regeneration were determined by BET analysis.^27^ Nitrogen adsorption–desorption tests were carried out simultaneously, and the corresponding isotherms are shown in Fig. 8 to investigate the structural changes of the catalyst before and after regeneration. Both samples exhibit typical type IV adsorption–desorption isotherms with obvious hysteresis loops when the relative pressure *P*/*P*_0_ is greater than 0.4, indicating the presence of mesoporous structures. For more measurement parameters of BET, please refer to the SI Table S1 and Fig. S1.

The nitrogen adsorption capacity of the deactivated catalyst is much lower than that of the regenerated catalyst in all relative pressure ranges, which indicates that the deposited pollutants partially block the pores during long-term operation. The relatively narrower hysteresis loop of the deactivated sample also reflects limited pore accessibility and decreased pore connectivity. The adsorption capacity is significantly improved over the entire pressure range after regeneration, and the hysteresis loop becomes more pronounced. The substances deposited in the pores are effectively removed, the blocked pores are reopened, and the mesoporous structure is reconstructed, which is consistent with these changes and results. The improved pore connectivity can facilitate the mass transfer process of VOC molecules and optimize the adsorption.

**Fig. 9:**
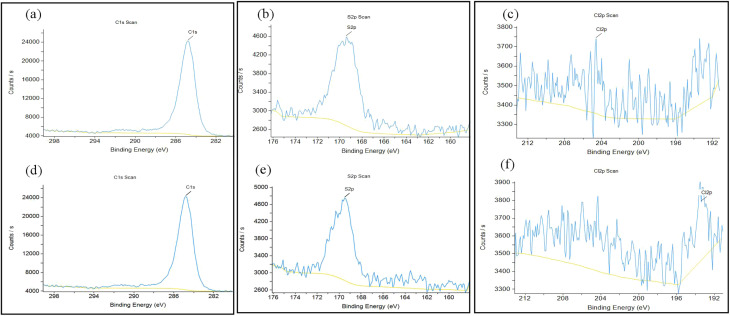
XPS characterization of the spent and regenerated catalysts: (a) C 1s spectrum of the spent catalyst; (b) S 2p spectrum of the spent catalyst; (c) Cl 2p spectrum of the spent catalyst; (d) C 1s spectrum of the regenerated catalyst; (e) S 2p spectrum of the regenerated catalyst; (f) Cl 2p spectrum of the regenerated catalyst.

*T*-plot analysis based on the Harkins–Jura model was used to further detect the microporous structure, and the relevant results are presented in Fig. 9. The intercept of the linear fitting interval of the regenerated catalyst is more positive, indicating that the permanent microporous structure within the carbon framework has been restored. The repaired microporous structure provides additional adsorption sites and improves catalytic performance. Table 1 summarizes the relevant structural parameters. After regeneration treatment, the BET specific surface area increases from 29.57 m^2^ g^−1^ to 54.44 m^2^ g^−1^, with an increase of approximately 84%. The microporous specific surface area rises from 11.85 m^2^ g^−1^ to 25.90 m^2^ g^−1^, and the total pore volume also increases from 0.0351 cm^3^ g^−1^ to 0.0510 cm^3^ g^−1^. The micropore volume is increased by approximately 69%, and the internal pore structure is significantly improved.

**Table 1:** Surface area and pore structure parameters of the catalyst before and after regeneration

Parameter	Before regeneration	After regeneration
Sample mass/g	0.083	0.087
BET surface area/(m^2^ g^−1^)	29.569	54.436
Micropore area (*t*-plot)/(m^2^ g^−1^)	11.847	25.901
External surface area (*t*-plot)/(m^2^ g^−1^)	17.722	28.534
Total pore volume/(cm^3^ g^−1^)	0.0351	0.051
Micropore volume (*t*-plot)/(cm^3^ g^−1^)	0.0076	0.0130
Mesopore volume (BJH)/(cm^3^ g^−1^)	0.0346	0.0415

The multi-step regeneration strategy includes physical cleaning, chemical cleaning and heat treatment processes, which can effectively remove surface contaminants and reopen blocked micropores and mesopores, and relevant experimental results verify this conclusion. The reconstructed hierarchical pore structure significantly improved pore accessibility and exposed more active sites, providing a structural basis for the recovery of catalytic activity.

#### 3.3.5 Thermogravimetric analysis

**Fig. 10:**
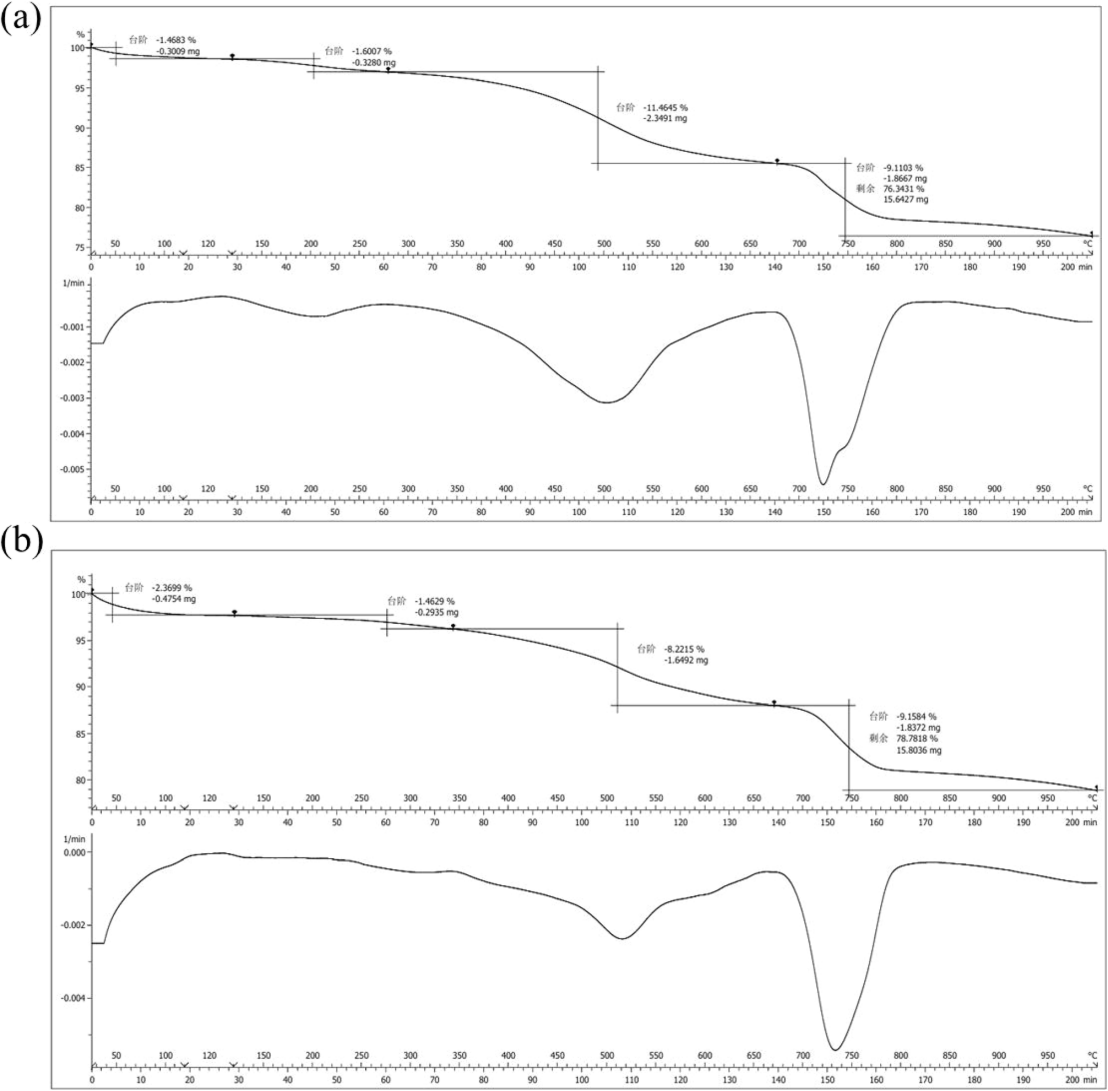
Thermogravimetric analysis of the catalysts before (a) and after (b) regeneration.

Thermogravimetric analysis (TGA) was conducted to investigate the thermal stability and evolution of deposited species on the activated carbon honeycomb catalysts before and after regeneration. As shown in Fig. 10, both samples have undergone several rounds of weight loss and contain different types of volatile and carbonaceous materials.

For the deactivated catalyst (Fig. 10(a)), a small weight loss below about 200 °C is due to the desorption of physically adsorbed water and weakly bound volatile substances. Subsequently, a gradual mass decrease between 200 and 500 °C is observed; this is mainly due to the decomposition of adsorbed organic compounds and oxygen-containing surface species that have accumulated over a long period of VOC removal operation. A relatively large weight-loss region occurs around 500–750 °C, which is associated with the oxidation and decomposition of stable carbonaceous deposits or strongly adsorbed organic residues. A relatively large exothermic peak in the DTG curve at around 700–750 °C is consistent with the combustion of refractory carbon deposits. Regeneration (Fig. 10(b)) has reduced the extent of overall weight loss. The first weight loss below 200 °C is smaller than that of the deactivated catalyst; therefore, it is assumed that loosely adsorbed water and other volatile substances have been removed during regeneration. In addition, the weight loss after high-temperature oxidation is about 11.45% for a deactivated catalyst and drops to about 8.22% for a regenerated catalyst in the 500–750 °C range; thus, some of the accumulated carbonaceous deposits were effectively removed during regeneration. The DTG profiles also show that both catalysts have a main oxidation peak in the range of 700–750 °C, which is due to the combustion of carbon materials. However, the regenerated catalyst has shown a reduced oxidation intensity; thus, it is assumed that the amount of residual combustible deposits is smaller and the surface cleaner after regeneration.

As shown by TGA, the regeneration treatment has reduced the quantity of deposited organic contaminants and carbonaceous species from VOC adsorption to a certain extent. Based on the above analysis, it can be inferred that partial pore reopening and a recovery of accessible pore structure have occurred. In addition, the reduction in the amount of surface deposits after regeneration may have exposed some adsorption sites for use; however, whether there is an increase in activity needs to be confirmed by further catalytic performance tests.

### 3.4 Regeneration mechanism analysis

**Fig. 11:**
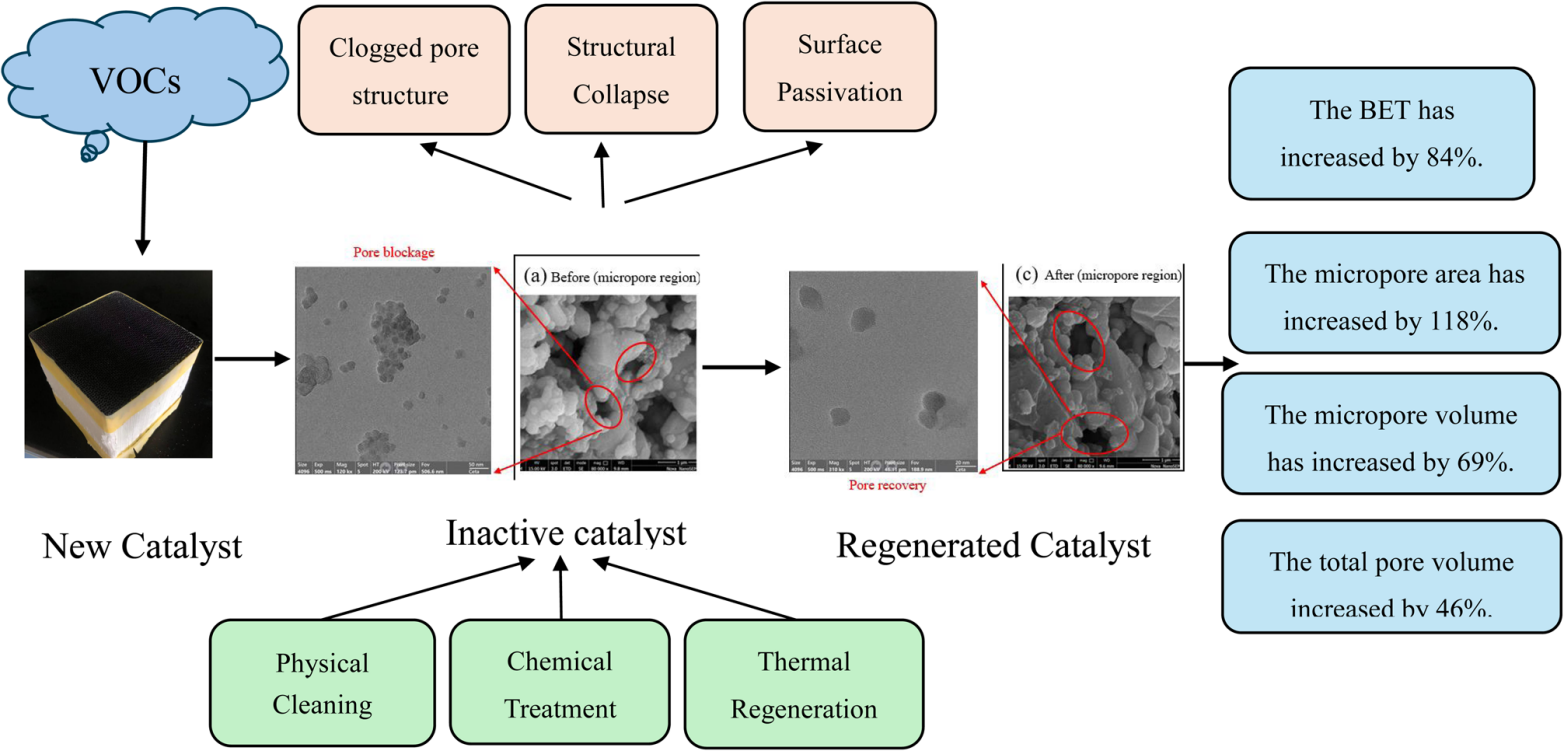
Catalyst regeneration mechanism diagram.

Based on the structural characterization results obtained from SEM, BET, and XRD analyses, a deactivation–regeneration mechanism of the VOCs activated carbon honeycomb catalyst was proposed, as illustrated in Fig. 11.

Throughout long-term industrial operation, VOC molecules were initially adsorbed on the catalyst surface and inside the pore channels. Under continuous reaction conditions, some adsorbed organic species underwent incomplete oxidation, polymerization, and carbonization reactions, resulting in the formation of carbonaceous deposits. Meanwhile, inorganic impurities such as sulfate, chloride, and metal-containing particulates gradually accumulated on the catalyst surface. These deposited species progressively blocked the pore channels, reduced pore accessibility, and covered active sites, ultimately leading to catalyst deactivation. At the physical cleaning stage, loosely attached dust particles and surface contaminants were partially removed by compressed air blowing and water washing. This pretreatment reduced external surface contamination and improved the accessibility of macroporous channels; however, carbonaceous deposits and inorganic salts inside the microporous structure remained partially retained. After chemical treatment, soluble inorganic contaminants attached to the surface and pores of the catalyst are effectively removed. At the end of the chemical cleaning stage, sulfate and nitrate deposits gradually dissolve, and the surface passivation layer is also partially removed. The partially blocked pores are re-opened, significantly improving the accessibility of the active sites inside the catalyst. In the thermal treatment stage, residual carbon deposits inside the catalyst are converted into gaseous products through oxidation and decomposition reactions at elevated temperatures. These products are then released from the catalyst surface. As the deposited carbon is removed, the blocked pore structure is gradually restored, improving access to active sites. The characterization results are consistent with this regeneration process. Compared with the deactivated catalyst, the regenerated catalyst shows increases of about 84% in BET surface area, 118% in micropore area, and 69% in micropore volume. SEM analysis confirms the removal of surface deposits and the reopening of pore channels. XRD patterns show no obvious changes in the carbon support or active phase structure after regeneration. Whether the catalyst performance can be restored is mainly related to several aspects. Firstly, the pollutants are removed, then the pores are reopened, and secondly, the structure itself can remain stable. When these effects are combined, they have a synergistic effect. The reconstructed layered pore structure not only greatly improves mass transfer efficiency, but also exposes the active sites again, thus restoring the adsorption and catalytic performance of the catalyst.

## 4. Conclusion

In this study, a multi-step regeneration strategy involving physical cleaning, chemical washing, and thermal treatment was developed for deactivated activated carbon honeycomb catalysts used in industrial VOC removal. The deactivation process was mainly associated with the accumulation of carbonaceous deposits, inorganic contaminants, and pore blockage during long-term operation.

Comprehensive characterization results demonstrated that regeneration effectively modified the structural and chemical properties of the catalyst. BET analysis revealed that the specific surface area increased from 29.57 to 54.44 m^2^ g^−1^, with significant recovery of microporous characteristics. SEM and TEM analyses confirmed the removal of surface deposits and partial reopening of blocked pore channels. XRD results indicated that the carbon framework and crystalline phases remained stable after regeneration, although thermal treatment induced changes in crystallite structure.XPS analysis further demonstrated that regeneration altered the surface chemical environment and partially removed sulfur- and chlorine-containing contaminants without destroying the primary carbon framework. TGA results confirmed that thermally stable carbonaceous residues were reduced after regeneration, providing direct evidence for the removal of accumulated organic deposits.

Based on the combined evidence from SEM, TEM, BET, XRD, XPS, and TGA analyses, the deactivation – regeneration mechanism was attributed to pollutant accumulation, pore blockage, surface contamination, and subsequent structural and chemical reconstruction during regeneration. Although the regenerated catalyst showed significant recovery of accessible pore structure, complete restoration to the original fresh catalyst state requires further long-term catalytic performance evaluation. These findings provide valuable insights into the regeneration of industrial activated carbon honeycomb catalysts and contribute to the development of sustainable VOC control technologies.

## Conflicts of interest

The authors declare that they have no known competing financial interests or personal relationships that could have appeared to influence the work reported in this paper.

## Supplementary Material

RA-OLF-D6RA04608H-s001

## Data Availability

The authors confirm that the data and material supporting the findings of this study are available in the article. Supplementary information (SI) is available. See DOI: https://doi.org/10.1039/d6ra04608h.
