# Supplementary material for: Regeneration of deactivated activated carbon honeycomb catalysts for VOC removal: structural evolution and activity recovery mechanisms
Source: RSC Adv. 2026 Jul 22. Online ahead of print. doi: 10.1039/d6ra04608h (PMC13390682; doi:10.1039/d6ra04608h)
Supplement: RA-OLF-D6RA04608H-s001 [file RA-OLF-D6RA04608H-s001.pdf]

Supplementary Information (SI) for RSC Advances.
This journal is © The Royal Society of Chemistry 2026

Supporting information

ASAP 2020 Plus 2.00 | ASAP 2020 V4.00 Serial # 1694 Unit 1 | Page 14 of 47

Sample: NJYC-2-QK#14
Operator:
Submitter:
File: C:\Users\60200\Desktop\YT\data1\20\NJYC-2.SMP

Started:	2026/3/18 14:14:22	Analysis adsorptive:	N2
Completed:	2026/3/18 16:07:39	Analysis bath temp.:	-195.759 °C
Report time:	2026/3/18 23:04:57	Thermal correction:	No
Sample mass:	0.0832 g	Ambient free space:	17.7459 cm³ Entered
Analysis free space:	46.0800 cm³	Equilibration interval:	5 s
Low pressure dose:	10.0000 cm³/g STP	Sample density:	1.000 g/cm³
Automatic degas:	Yes		

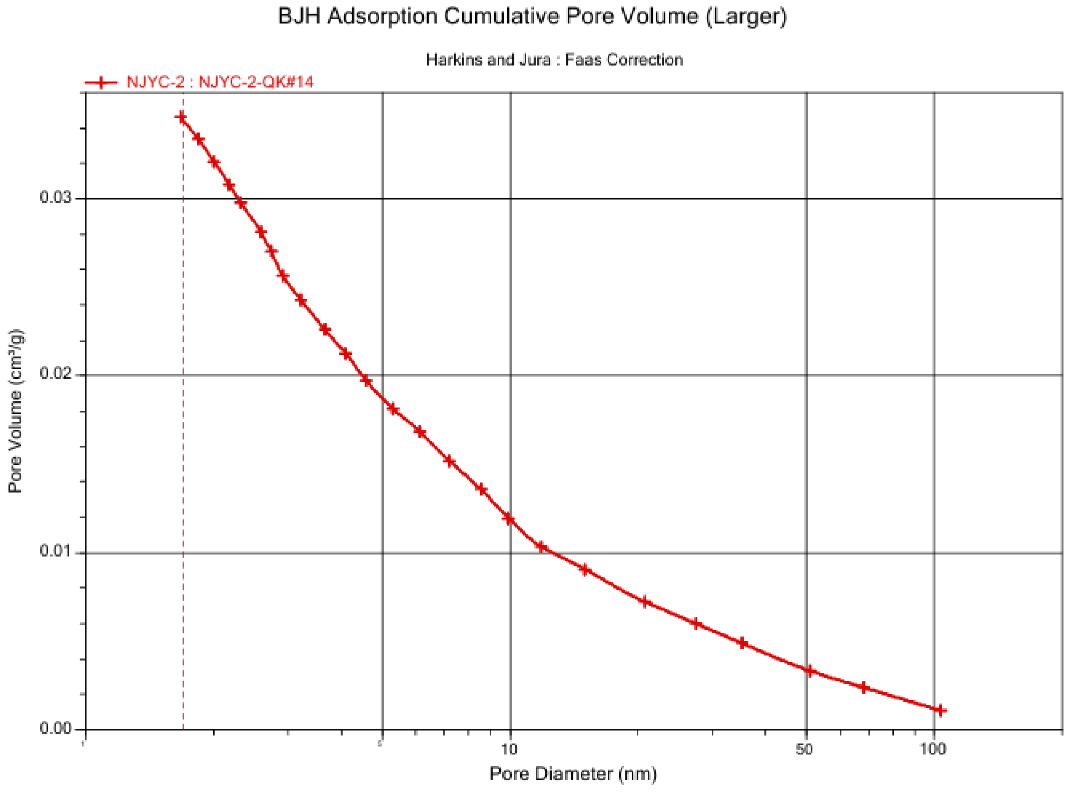

Figure S1. BJH Adsorption Cumulative Pore Volume of before regeneration.

Sample: NJYC-1-QK#12
Operator:
Submitter:
File: C:\Users\60200\Desktop\YT\data1\20\NJYC-1.SMP

Started: 2026/3/18 7:34:25
Completed: 2026/3/18 9:54:05
Report time: 2026/3/18 23:04:48
Sample mass: 0.0872 g
Analysis free space: 48.7111 cm³
Low pressure dose: 10.0000 cm³/g STP
Automatic degas: Yes

Analysis adsorptive: N2
Analysis bath temp.: -195.761 °C
Thermal correction: No
Ambient free space: 18.0727 cm³ Entered
Equilibration interval: 5 s
Sample density: 1.000 g/cm³

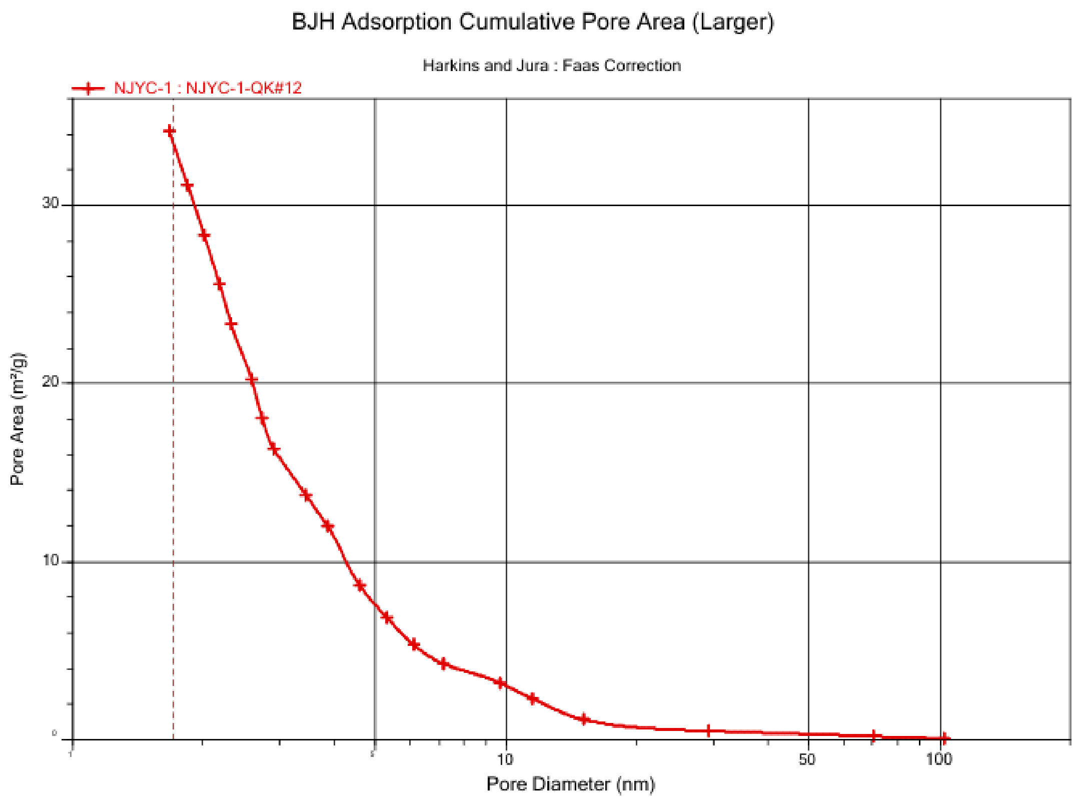

Figure S2. BJH Adsorption Cumulative Pore Volume of after regeneration.

Table S1. BET measurement parameters of the catalyst before and after regeneration.

Parameter	Before Regeneration	After Regeneration
Adsorptive	N_2	N_2
Analysis bath temperature /°C	-195.761	-195.759
BET correlation coefficient	0.99995	0.99965
Standard deviation of fit / (cm^3 g^{-1} STP)	0.12512	0.07513
